# Superior protective effects of PGE2 priming mesenchymal stem cells against LPS-induced acute lung injury (ALI) through macrophage immunomodulation

**DOI:** 10.1186/s13287-023-03277-9

**Published:** 2023-03-22

**Authors:** Kamal Hezam, Chen Wang, Enze Fu, Manqian Zhou, Yue Liu, Hui Wang, Lihong Zhu, Zhibo Han, Zhong-Chao Han, Ying Chang, Zongjin Li

**Affiliations:** 1 Nankai University School of Medicine, Tianjin, 300071 China; 2 Tianjin Key Laboratory of Human Development and Reproductive Regulation, Tianjin Central Hospital of Gynecology Obstetrics, Nankai University Affiliated Hospital of Obstetrics and Gynecology, Tianjin, 300052 China; 3 The Key Laboratory of Bioactive Materials, Ministry of Education, Nankai University, College of Life Sciences, Tianjin, 300071 China; 4 Department of Radiation Oncology, Tianjin Union Medical Center, Nankai University, Tianjin, 300120 China; 5 Department of Gynecologic Oncology, Beijing Obstetrics and Gynecology Hospital, Capital Medical University, Beijing, 100026 China; 6 Jiangxi Engineering Research Center for Stem Cells, Shangrao, 334109 Jiangxi China; 7 Tianjin Key Laboratory of Engineering Technologies for Cell Pharmaceuticals, National Engineering Research Center of Cell Products, AmCellGene Co., Ltd, Tianjin, 300457 China; 8 Beijing Engineering Laboratory of Perinatal Stem Cells, Beijing Institute of Health and Stem Cells, Health & Biotech Co., 100176 Beijing, China; 9 State Key Laboratory of Kidney Diseases, Chinese PLA General Hospital, Beijing, 100853 China

**Keywords:** Acute lung injury (ALI), Acute respiratory distress syndrome (ARDS), Prostaglandin E2 (PGE2), Mesenchymal stem cells (MSCs), Priming

## Abstract

**Background:**

Mesenchymal stem cells (MSCs) have demonstrated remarkable therapeutic promise for acute lung injury (ALI) and its severe form, acute respiratory distress syndrome (ARDS). MSC secretomes contain various immunoregulatory mediators that modulate both innate and adaptive immune responses. Priming MSCs has been widely considered to boost their therapeutic efficacy for a variety of diseases. Prostaglandin E2 (PGE2) plays a vital role in physiological processes that mediate the regeneration of injured organs.

**Methods:**

This work utilized PGE2 to prime MSCs and investigated their therapeutic potential in ALI models. MSCs were obtained from human placental tissue. MSCs were transduced with firefly luciferase (Fluc)/eGFP fusion protein for real-time monitoring of MSC migration. Comprehensive genomic analyses explored the therapeutic effects and molecular mechanisms of PGE2-primed MSCs in LPS-induced ALI models.

**Results:**

Our results demonstrated that PGE2-MSCs effectively ameliorated lung injury and decreased total cell numbers, neutrophils, macrophages, and protein levels in bronchoalveolar lavage fluid (BALF). Meanwhile, treating ALI mice with PGE2-MSCs dramatically reduced histopathological changes and proinflammatory cytokines while increasing anti-inflammatory cytokines. Furthermore, our findings supported that PGE2 priming improved the therapeutic efficacy of MSCs through M2 macrophage polarization.

**Conclusion:**

PGE2-MSC therapy significantly reduced the severity of LPS-induced ALI in mice by modulating macrophage polarization and cytokine production. This strategy boosts the therapeutic efficacy of MSCs in cell-based ALI therapy.

**Supplementary Information:**

The online version contains supplementary material available at 10.1186/s13287-023-03277-9.

## Introduction

Acute lung injury (ALI) and its subsequent form acute respiratory distress syndrome (ARDS) are important causes of morbidity and mortality worldwide. They have become one of the most global burdens in the twenty-first century due to coronavirus disease 2019 (COVID-19) [1, 2]. ALI occurs due to massive inflammatory processes that cause epithelial and endothelial lung injury, leading to increased vascular permeability. Furthermore, several reports show that macrophages, neutrophils, and their related factors play crucial roles in lung inflammation [3, 4]. Therefore, there is an urgent need to find an effective therapeutic to target these abnormalities, which could be an effective strategy to prevent and treat ALI.

Mesenchymal stem cells (MSCs) are multipotent progenitor cells that can differentiate into numerous cell types and are present in several tissues [5–7]. Increasing evidence of MSC migration to lung injury and their contribution to lung regeneration attracts attention for the treatment of ALI/ARDS [8]. For these reasons, the use of MSCs in preclinical and clinical settings has been extensively investigated. In animal and human lung perfusion models, both intravenous and intratracheal administration of MSCs for ALI significantly improved alveolar permeability and inflammation [9, 10]. Various paracrine factors produced by MSCs play a crucial role in influencing the microenvironment of injured tissues and modulating the immune response. These factors are transforming growth factor (TGF)-β1, hepatocyte growth factor (HGF), prostaglandin E2 (PGE2), interleukin-6 (IL-6), interleukin (IL-10), and nitric oxide (NO) [11, 12]. Consequently, therapeutic applications of MSCs remain promising for ALI/ARDS.

Prostaglandin E2 (PGE2) is a lipid signaling molecule that plays an important role in the modulation of inflammatory and fibrotic diseases [13, 14]. It can be synthesized by many tissue cells, such as epithelial cells, fibroblasts, and inflammatory cells, that infiltrate tissues after partial injury [15–18]. By interacting with the E-type prostaglandin receptor (EP) family, it plays a role in a wide range of physiological processes and, as a result, facilitates the regeneration of multiple organ systems following injury. The production of PGE2 in damaged tissues is significantly increased, and many studies have reported PGE2-regulated roles in activating endogenous stem cells, the immune response, angiogenesis, and other processes [19, 20]. Due to these functions, PGE2 was hypothesized and selected to strengthen the protective effects of MSCs against LPS-induced ALI models.

Macrophages are considered a key component of the innate immune system. Due to their plasticity, macrophages play a very important role in tissue regeneration. They have been divided into two categories, classically activated and proinflammatory M1 macrophages or alternatively activated and anti-inflammatory M2 macrophages [21–23]. The role of MSCs and their secretomes in macrophage immunomodulation has been studied in a wide range of models. Previous studies reported that MSCs play a critical role in mediating macrophage polarization from the proinflammatory (M1) form to the anti-inflammatory (M2) form by regulating the production of cytokines such as IL-10, IL-1β, IL-6 and TNF-α [18, 24–26]. Hence, regulating macrophage polarization is critical for lung repair and regeneration.

In recent years, the priming strategy has been considered to stimulate and improve the therapeutic effects of MSCs. Different stimuli have previously been used, including cytokines, hypoxia, biochemical factors, and biomaterials [27, 28]. The lipid signaling molecule prostaglandin E2 (PGE2), an inflammatory mediator, can enhance tissue regeneration and repair after injury in various organ systems [19, 29]. In this study, we used PGE2 to prime MSCs and investigated the therapeutic potential of PGE2-MSCs for acute lung injury. Furthermore, we highlighted the antifibrotic effects and possible mechanisms of PGE2-MSCs in ALI. We found that PGE2-MSCs significantly alleviated ALI and mediated lung regeneration. We also explored whether macrophage polarization plays an essential role in the anti-inflammatory effect of PGE2-MSCs in ALI mice.

## Materials and methods

### Animals

ALI models were established as previously reported [30, 31]. In summary, 2.5% avertin was administered intraperitoneally to anesthetize C57BL/6 mice (8–10 weeks old, weighing 22–25 g), and lipopolysaccharide (LPS, O55:B5; 5 mg/kg; Sigma-Aldrich, dissolved in PBS) was administered intratracheally to cause lung injury. Then, intravenously administered MSCs and PGE2-MSCs were applied 6 h later. The International Guiding Principles for Biomedical Research Involving Animals, which the Council published for the International Organizations of Medical Sciences, were followed by all experiments, which were approved by the Nankai University Animal Care and Institutional Animal Care Committees (approval no. 20170022).

### Experimental protocol

C57BL/6 mice (female, 8–10 weeks old, weighing 22–25 g) were randomly divided into five groups: sham group, ALI group (LPS only, 5 mg/kg), PBS group, MSC group (1 $\times$ 10^6^) and PGE2-MSCs group (1 $\times$ 10^6^). The mice were anesthetized with inhaled isoflurane (2% to 3%), and then LPS (5 mg/kg) was administered intratracheally to induce lung injury. After 6 h of LPS administration, the ALI mice were intravenously injected with 250 µl of PBS, which was used as a solvent control as previously described [32]. At different time points, survival rates and body weight ratios were calculated. Then, bronchoalveolar lavage fluid (BALF) and lung tissue samples were obtained and used for hematoxylin and eosin (H&E) staining, quantitative real-time polymerase chain reaction (qRT‒PCR), Western blot assay (WB), etc.

### Cell culture

As previously reported [33, 34], human placental MSCs (hP-MSCs) were cultured in DMEM/F12 media with 10% fetal bovine serum (FBS) (Australia), 1% NEAA (Gibco), 1% L-glutamine (Gibco), and 1% penicillin and streptomycin (Gibco). MSCs were transduced with a self-inactivating lentiviral vector containing a ubiquitin promoter driving firefly luciferase and an enhanced green fluorescence protein (Fluc-eGFP) double fusion (DF) reporter gene to monitor transplanted cells in vivo [6, 32]. MSCs were incubated at 37 °C in a humidified incubator containing 5% CO_2_. At the beginning of the experiment, PGE2 (CAS 363–24-6; Santa Cruz Biotechnology) was added to the culture medium of MSCs. The final concentration was 2 µmol/L. After 12 h of culture, cells were washed and resuspended with PBS prepared for mice injection. The total collected cell was 1 × 10^6^ cells, and the route of administration was intravenous.

### Bioluminescence imaging of Fluc-eGFP-labeled MSCs

For bioluminescence imaging (BLI), firefly luciferase (Fluc) was used for MSCs as previously described [35, 36]. For intravital imaging, ALI mice were established by intratracheal injection of LPS. After that, ALI models received intravenous injections of Fluc-labeled 1 × 10^6^ total MSC cells in a volume of 250 μL. After 24 h, ALI mice were imaged using the IVIS Lumina Imaging System (Xenogen Corporation, Hopkinto, MA) after intraperitoneal injection of the substrate of D-luciferin (150 mg/kg; Biosynth International, USA).

### Cell counting and protein concentration assay of BALF

To collect BALF, all mice were euthanized after LPS challenge and treatment with PGE2-MSCs and MSCs. The BALF samples were centrifuged to pellet the cells, washed twice with ice-cold PBS and collected before being centrifuged for 5 min at 4 °C. Next, the sedimented cells were resuspended in PBS to obtain total cell counts using a hemocytometer. Neutrophils and macrophages were counted using the Wright-Giemsa staining method [37].

### Histopathological evaluation

The left and right upper lobes (RUL) were collected from all groups that were not subjected to BALF collection. The lung tissue samples were fixed for 48 h in 4% PFA, dehydrated in a series of graded ethanol, embedded in paraffin wax, and cut into 5-μm-thick sections. The paraffin-embedded sections were stained with hematoxylin and eosin (H&E) for pathological analysis.

### Immunofluorescence staining

Tissue samples from the lungs were fixed in 4% PFA and dehydrated in 30% sucrose solution before being embedded in (OCT) (Sakura Finetek, 4583, Japan). All samples were cut into 5 µm sections transversely. Anti-rabbit F4/80 antibodies (28,463–1-AP, Proteintech) and anti-rabbit CD206 antibodies (Abcam) were used to stain the samples (18,704–1-AP, Proteintech). The nuclei of each sample were stained with 4′,6-diamidino-2-phenylindole (DAPI, C0065, Solarbio), and all samples on glass slides were mounted with mounting medium and antifade (Solarbio, S2100). Fluorescence images were captured using an Olympus fluorescence microscope. Complete details on antibodies can be found in Additional file 1: Table S1.

### Quantitative real-time PCR

Total RNA was isolated from the cells and tissues using TRIzol reagent (Takara, Japan) according to the manufacturer’s instructions. cDNA was generated based on TransScript Fly First-Strand cDNA Synthesis SuperMix (YEASEN, China). qRT‒PCR was performed using SYBR Green PCR Master Mix (YEASEN, China). A CFX96 TM Real-Time PCR System was used for the real-time PCR analysis (Bio-Rad, USA). The estimate of gene expression was normalized to actin expression and calculated using the 2(^−ΔΔCt^) method. The primer sequences can be found in Additional file 1: Table S2.

### Western blot analysis

Tissues prepared for western blotting were lysed in radioimmunoprecipitation assay (RIPA) buffer (Solarbio, Shanghai, China). The protein concentration was measured using a BCA protein assay kit (GenStrar, China). A total of 30 µg of protein from each sample was run on 10% SDS‒PAGE gels in electrophoresis buffer and transferred to polyvinylidene fluoride membranes (PVDF; Millipore, Darmstadt, Germany). Then, skim milk (5%) was used as a blocking buffer for 2 h, and the membrane was incubated with primary antibodies overnight at 4 °C. After washing, the membrane was incubated with horseradish peroxidase (HRP)-conjugated secondary antibodies for 2 h. Signals were visualized with a Pierce-enhanced chemiluminescence Western blotting substrate (Millipore). GAPDH was used as the loading control. Complete details on antibodies can be found in Additional file 1: Table S1.

### RNA sequencing

Total RNA samples from MSCs and PGE2-MSCs were isolated with Trizol (Invitrogen) for RNA sequencing (RNA-seq). Three samples from each group were harvested for RNA isolation. RNA quantity and quality were determined using a Nanodrop (Thermo Scientific, Waltham, MA). RNA-seq was performed using the RNA Nano 6000 Assay Kit of the Agilent Bioanalyzer 2100 system (Agilent Technologies, CA). Gene ontology (GO) classification and distribution analysis of gene function were done with the Gene Ontology Consortium (Gene Ontology http://geneontology.org/). Gene set enrichment analysis (GSEA, www.broadinstitute.org/gsea) was performed. The Kyoto Encyclopedia of Genes and Genomes (http://www.kegg.jp/) database was used for the genome information and system functions analysis.

### Statistical analysis

All data are shown as the mean ± SEM. of at least three independent replicates. GraphPad Prism version 5.01 was used to analyze all statistical comparisons one-way for multigroup comparisons (GraphPad Software, San Diego, CA, USA). Asterisks denote statistical significance in each figure and the reference group. The basis for comparison was displayed in plus sign if needed.

## Results

### Characterization and biodistribution of PGE2-MSCs and MSCs

**Fig. 1:**
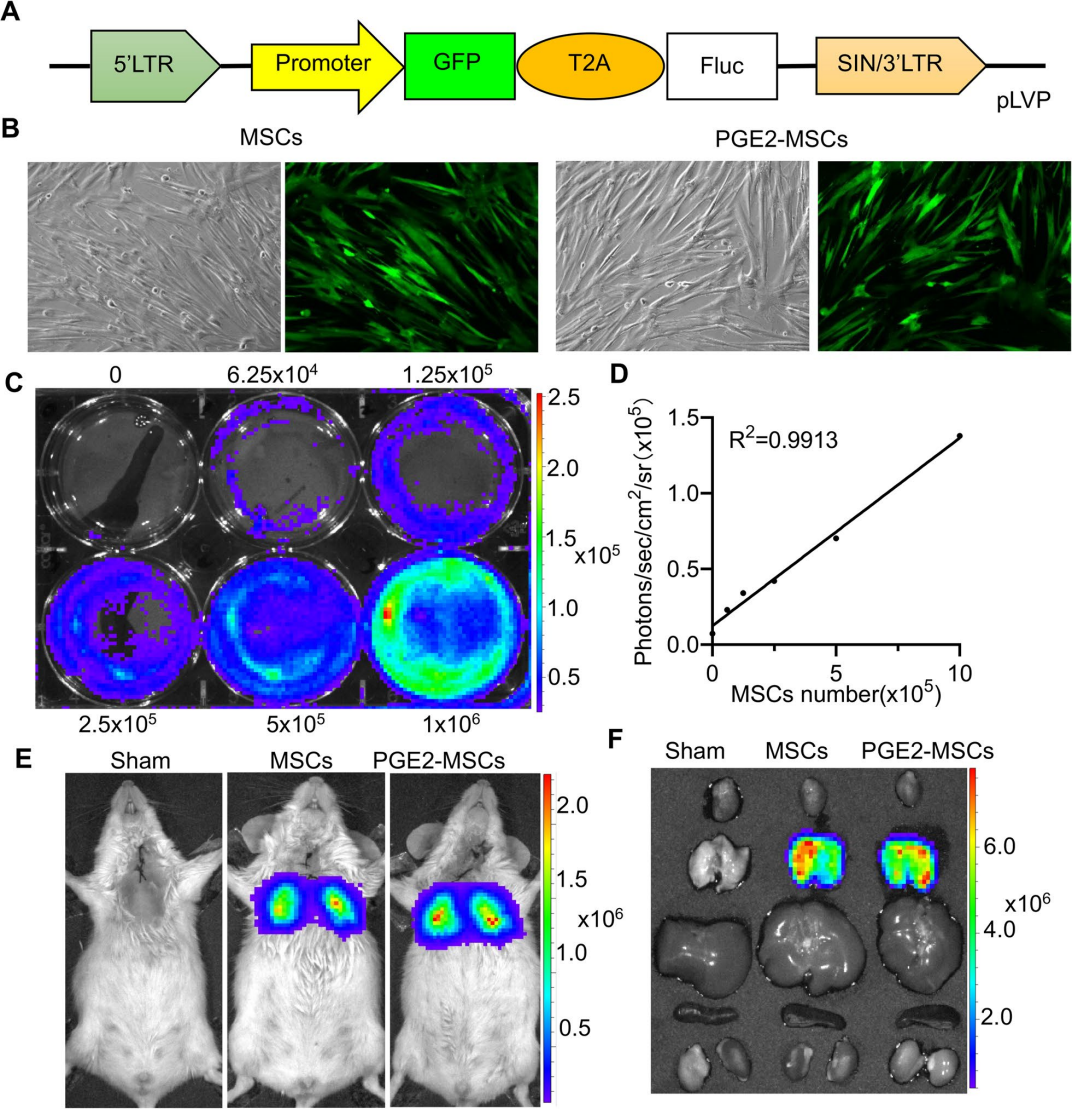
Characterization and biodistribution of PGE2-MSCs and MSCs. **A** Schematic representation of the double-fusion (DF) firefly luciferase (Fluc) and green fluorescent protein reporter gene containing Fluc and GFP driven by ubiquitin promotion. **B** Brightfield and fluorescence microscopy showing GFP expression in MSCs and PGE2-MSCs. **C, D** The quantification BLI of MSCs and PGE2-MSCs shows a robust correlation between cell number and Fluc activity**.** The signal activity was represented by photons/s/cm^2^/steradian. **E, F** Fluc signals were tracked by BLI analysis to monitor the retention of transplanted PGE2-MSCs and MSCs in vivo. The signal activity was represented by photons/s/cm^2^/steradian

To monitor PGE2-MSCs in vivo in real time, DFMSCs were transduced into MSCs for labeling and injected intravenously (Fig. 1A–D). BLI analysis revealed a significant linear correlation between the amount of PGE2-MSCs and Fluc activity. The determination of the concentration of LPS to establish ALI models has been achieved using different doses of LPS for various animal models. Based on preliminary data, 5 mg/kg LPS was used for LPS-ALI models, and 2 µmol/L PGE2 was used as a priming suitable dose for MSCs culture (Additional file 1: Fig. S1A-B). We intraperitoneally injected Fluc substrate (D-luciferin; 150 mg/kg; Biosynth International, USA) at different time points. Average radiance quantified the BLI signal from the region of interest (ROI) in the lung after LPS-induced ALI (Fig. 1E,  F). The results revealed that the labeling and priming by PGE_2_ were specific for the targeting of the lung of LPS-induced ALI models and revealed therapeutic potential, encouraging further investigation.

### Treatment with PGE2-MSCs protects LPS-induced ALI mice

**Fig. 2:**
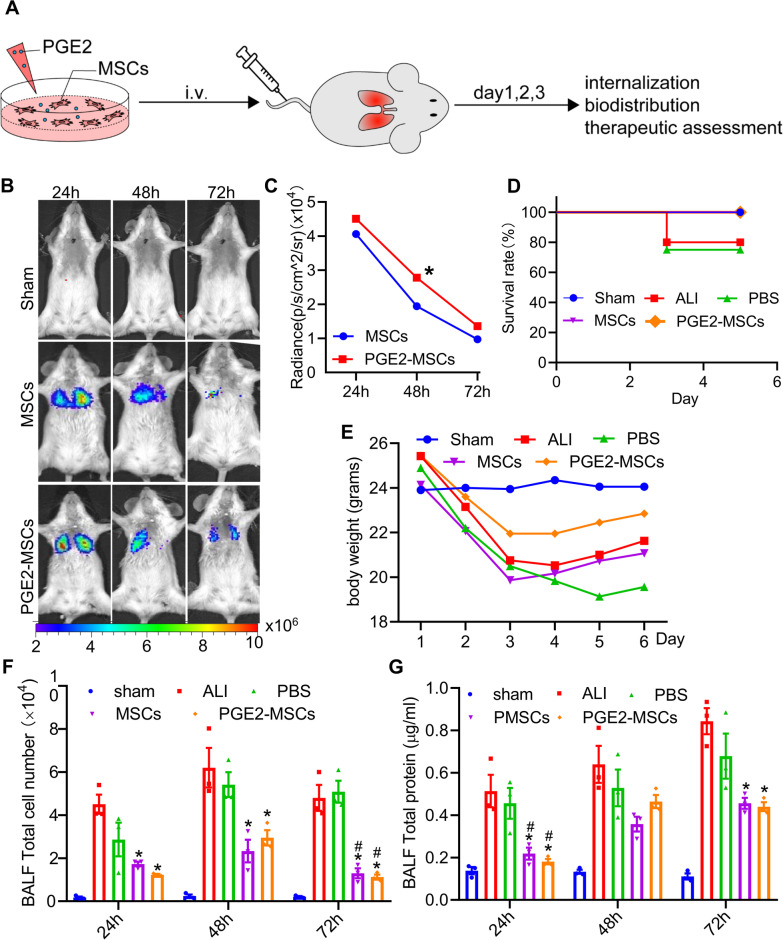
Protective effects of PGE2-MSC treatment against LPS-ALI in mice. **A** Schematic of the priming of PGE2-MSCs and study design. **B** Representative BLI of mice transplanted with PGE2-MSCs and MSCs for in vivo monitoring. **C** Quantitative analysis of BLI signals showed that the survival of PGE2-MSCs were better than those of MSCs. Data are expressed as the mean ± SEM. *P < 0.05. **D** Kaplan–Meier survival curves of LPS-induced ALI mice after administration of PGE2-MSCs, MSCs and PBS for 5 days. **E** Body weight was measured daily to observe the severity of ALI. **F, G** Total cell count and total protein concentration in BALF of LPS-induced ALI mice with or without MSC treatment at 24 h 48 h and 72 h. Data are presented as the mean ± SEM. *P < 0.05 vs ALI;^#^*P* < 0.05 vs PBS; ^&^*P* < 0.05 vs MSCs

ALI models and BALF samples were collected to investigate the therapeutic effects of (PGE2-MSCs and MSCs) on lung radiance, total cells, total protein concentration, survival rate, and weight rate as designed in Fig. 2A. First, as previously described, we used BLI to track PGE2-MSC and MSC survival in LPS-induced ALI models longitudinally. In summary, we observed the retention and stability of PGE2-MSCs and MSCs in vivo. A total of 1 $\times$ 10^6^ Fluc-labeled PGE2-MSCs suspended in PBS were intravenously injected into ALI mice at a total volume of 250 μL. At different times, mice were imaged immediately after intraperitoneal injection of D-luciferin (150 mg/kg) using the IVIS Lumina Imaging System. The BLI findings indicated that the robust Fluc signals in all groups indicated successful injection of PGE2-MSCs and MSCs, and the survival of PGE2-MSCs were better than those of MSCs, as shown in Fig. 2B, C. Our results also found that, based on Kaplan‒Meier survival curves, the highest overall survival rates were observed in the PGE2-MSC group compared with the control groups (Fig. 2 D). Furthermore, the PGE2-MSC-treated models improved the weight reduction of the LPS-ALI models compared to the control groups (Fig. 2E). Cell counting and protein levels were measured in BALF to analyze lung damage and inflammation. The total protein level and cell number increased in the ALI and PBS groups but were reduced in the PGE2-MSC and MSC groups (Fig. 2F, G). These results indicate that PGE2-MSCs may effectively reduce cellular infiltration as well as lung injury in ALI models.

### PGE2-MSCs affect lung tissue repair and the immune response

**Fig. 3:**
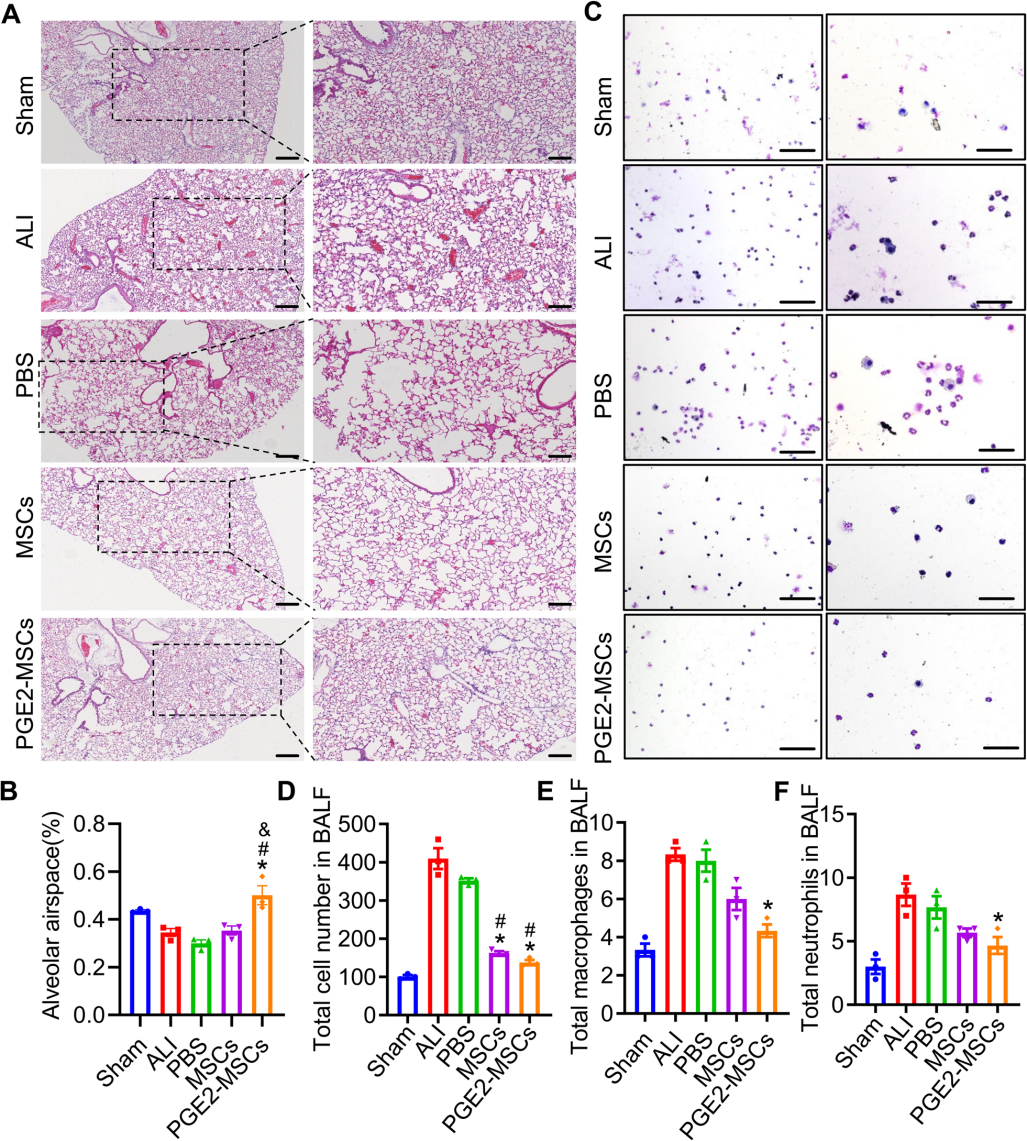
Effects of PGE2-MSC treatment on lung tissue repair and the immune response. **A, B** HE staining of the lungs of the mice (n = 5) of each experimental group was processed for histological evaluation 24 h after the LPS challenge, and the lung injury score was determined as a percentage. **C-F** Total cell counts per field, macrophages, and neutrophils from the BALF were counted using a hemocytometer, and the Wright-Giemsa staining method was used for cytosine staining (HE magnification is 100 × and 200x, the Wright-Giemsa is 40 × and 100x). Data are represented as the mean ± SEM. *P < 0.05 vs ALI;^#^*P* < 0.05 vs PBS; ^&^*P* < 0.05 vs MSCs

The protective effects of PGE2-MSCs against LPS-ALI have been explored in histopathological and immune cell profile evaluations. We assessed the therapeutic effects of PGE2-MSCs (Fig. 3A-F). The histopathological evaluation of lung tissues was examined with a light microscope. Our results showed that the normal structure of alveolar and interstitial tissues was destroyed in LPS-ALI mice, and inflammatory infiltration was prominent, while the PGE2-MSC-treated groups evidently improved the histopathological changes in the LPS-ALI models by reducing inflammatory cells and alveolar hemorrhage compared with the control groups. PGE2-MSCs also improved the lung injury score in LPS-ALI mice compared to the control groups (Fig. 3A, B). Furthermore, PGE2-MSC and MSC treatments remarkably reduced the total number of cells, macrophages, and neutrophils per field compared to the control treatments (Fig. 3C-F). Collectively, our findings found that PGE2-MSCs alleviated LPS-induced ALI.

### Identification of the potential mechanisms of PGE2-MSCs in LPS-ALI Mice by RNA-Seq

**Fig. 4:**
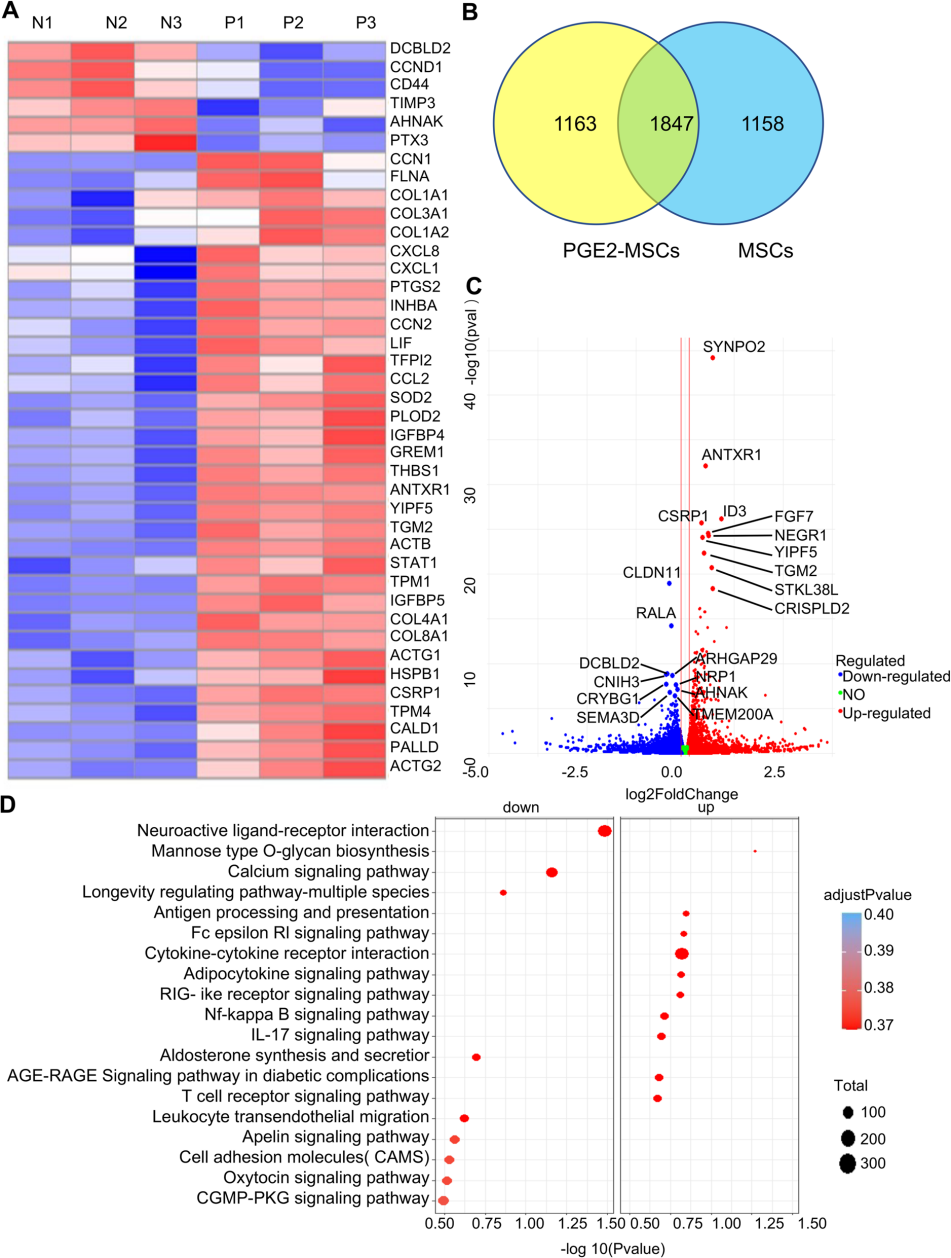
RNA analysis of PGE2-MSCs. **A** Heatmap of the RNA-seq analysis showing differences in the expression of the phagocytosis, anti-inflammation and fibrogenic components of MSCs primed with PGE2. N represents MSCs (without treatments), and P represents PGE2-MSCs. **B** Venn diagrams showing the overlap between genes from MSCs primed with PGE2 and untreated MSCs. **C** The volcano plot of differentially expressed genes was drawn by the ggplots2 software package. The volcano plot shows the distribution of genes, the fold difference of gene expression, and the significance results. **D** Dotplot pathway enrichment map showing the significantly overrepresented pathways

To further investigate the molecular mechanisms by which PGE2-MSCs attenuate LPS-ALI models, comprehensive RNA-seq analyses were performed for both PGE2-MSCs and MSCs. Cluster analysis was used to evaluate the expression patterns of differentially expressed genes under different experimental conditions; genes with high expression levels between samples were classified into different categories. These genes are involved in certain biological processes or in certain metabolic processes. There is a real connection in the signaling pathways. Therefore, we can discover unknown biological connections between genes by clustering expressions. We used a heatmap package to perform a two-way cluster analysis on the union and samples of different genes in all comparison groups, clustering according to the expression level of the same gene in different samples and the expression pattern of different genes in the same samples. Our results showed that the top 40 expressed genes were related to immune response processes such as macrophages and neutrophils and cytokine signaling (Fig. 4A).

To count the set of significantly differentially expressed genes and make a histogram of differentially expressed genes between other comparison groups, Venn diagram analyses were used to provide a comprehensive profile of PGE2-MSCs and MSCs. It also counts the number of differentially expressed genes that were upregulated and downregulated in each comparison group. In total, 1163 genes were significantly upregulated in PGE2-MSCs compared with MSCs, and most of them were related to functional biological processes, including regulation of cell proliferation, cytokine signaling, immune responses, and metabolic processes (Fig. 4B).

A volcano map was drawn using the ggplot2 package for mapping differentially expressed genes. The volcano map shows the distribution of genes, the difference in gene expression multiples, and the significant results. Under normal conditions, the distribution of differential genes on the left and right of the figure should be roughly symmetric, with the left side being Case compared to Control. The dot plot also represents an overview of pathway enrichment analysis for up-regulated genes and down regulated genes (Fig. 4C, D).

**Fig. 5:**
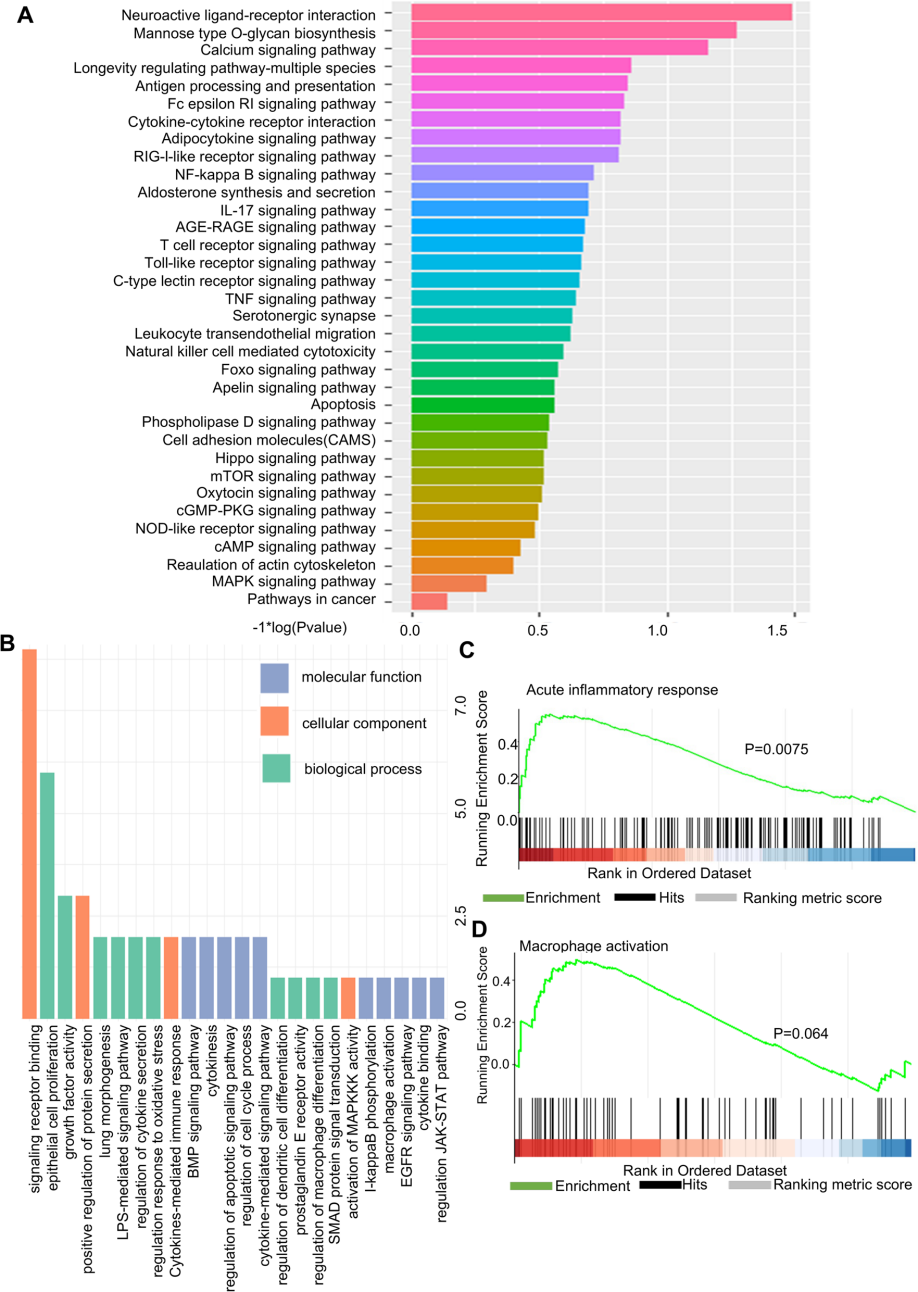
PGE2-primed MSCs revealed a unique protein expression profile. **A** Kyoto Encyclopedia of Genes and Genomes (KEGG) pathway analysis of PGE2-MSC protein expression. **B** GO analysis of proteins in PGE2-MSCs. **C, D** Gene set enrichment analysis (GSEA) of PGE2-MSCs, revealing the regulation of the acute inflammatory response and macrophage activation. The p value was calculated by GSEA

To understand the biological functions of the proteins contained in PGE2-MSCs and MSCs, we used gene ontology (GO) enrichment analysis and Kyoto Encyclopedia of Genes and Genomes (KEGG) pathway analysis of coexpressed proteins. Most of the factors identified were related to the cytokine signaling pathway, metabolic proteins and catabolic proteins (Fig. 5A,  B & Additional file 1: Fig. S2A-B). The enrichment analyses of the gene set (GSEA) of PGE2-MSCs-RNA-seq analysis indicated that transcription of genes associated with the acute inflammatory response and macrophage activation was significantly downregulated (Fig. 5C,  D). Taken together, these findings suggest that PGE2-MSCs positively regulate several signaling pathways with significant effects on macrophages and other immune cells. The general impact of this positive regulation could be responsible for lung amelioration and modulation of the immune response in LPS-ALI mice.

### Treatment with PGE2-MSCs attenuates LPS-induced ALI in mice through regulating macrophage polarization and cytokine production

**Fig. 6:**
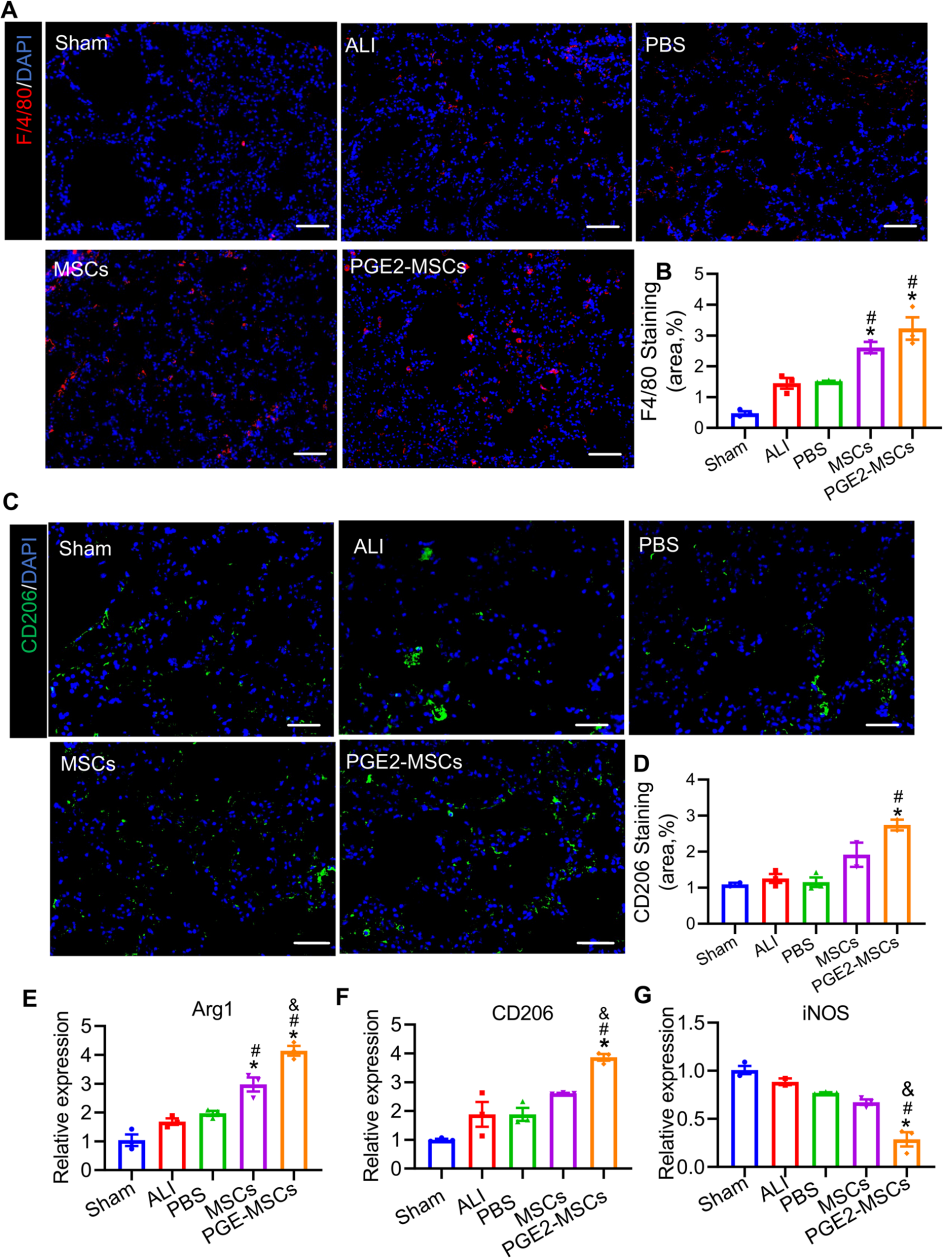
PGE2-MSCs regulate macrophage polarization and cytokine production. **A** Representative immunofluorescence images of F4/80 expression (red) in LPS-induced ALI mice. Scale bar, 200 µm. **B** Quantification of F4/80 + cells in each group. **C** Immunofluorescence images of CD206 expression (green) in LPS-induced ALI mice. Scale bar, 100 µm. **D** Quantification of CD206 + cells in each group. **E–G** qRT‒PCR analysis of macrophage-related gene expression (Arg1, CD206, IL-6 and iNOS). Data are represented as the mean ± SEM. *P < 0.05 vs ALI;^#^*P* < 0.05 vs PBS; ^&^*P* < 0.05 vs MSC

**Fig. 7:**
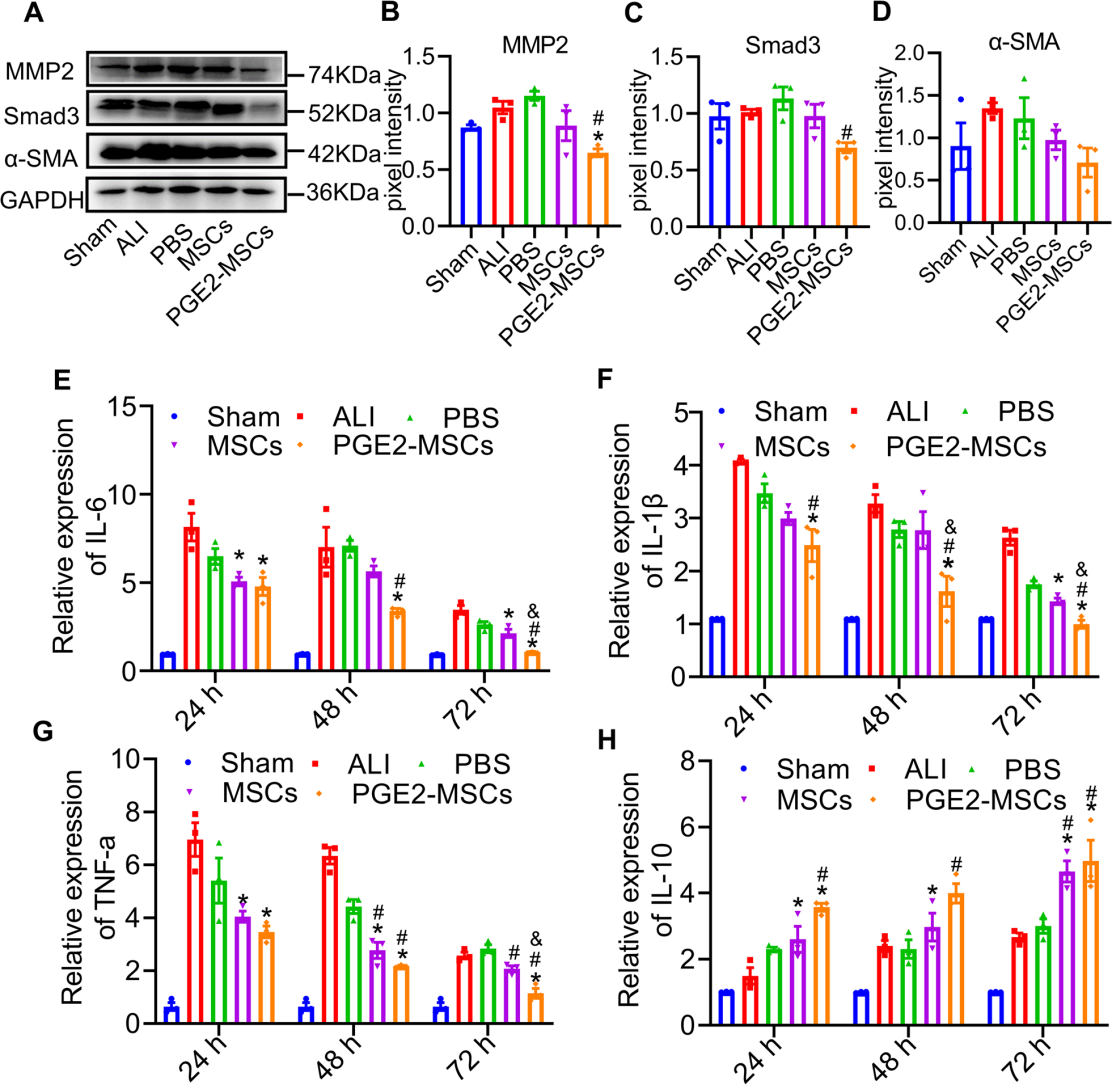
Therapeutic mechanisms of PGE2-MSCs in LPS-ALI mice **A-D** Representative immunoblot and expression analysis of SMAD3, MMP2, and α-SMA in LPS-induced ALI mice. **E** qRT‒PCR analysis of IL-6 mRNA in LPS-ALI mice monitored for 24 h, 48 h, and 72 h. **F** qRT‒PCR analysis of IL-1β mRNA in LPS-ALI mice monitored for 24 h, 48 h, and 72 h. **G** qRT‒PCR analysis of TNF-α mRNA in LPS-ALI mice monitored for 24 h, 48 h, and 72 h. **H** qRT‒PCR analysis of IL-10 mRNA in LPS-ALI mice monitored for 24 h, 48 h, and 72 h. Data are represented as the mean ± SEM. *P < 0.05 vs ALI;^#^*P* < 0.05 vs PBS; ^&^*P* < 0.05 vs MSC

To assess the potential mechanism of PGE2-MSCs and MSCs to protect LPS-induced ALI mice, we tested M1 and M2 polarization experimentally and supported these findings by complete analysis of RNA-Seq and immunofluorescence staining for F4/80 and CD 206, as illustrated in Fig. 6A-G & Additional file 1: Fig. S3. The same immunohistochemical results were also obtained for F4/80 (Fig. 6A, B) and CD 206 (Fig. 6C, D). By qRT‒PCR, the relative levels of the expression of the M2 polarization markers Arg-1 and CD 206 in the PGE2-MSC group were the highest compared to those in the other groups (Fig. 6E-G). In contrast, tumor necrosis factor-α (TNF-α), interleukin-6 (IL-6), interleukin-1β (IL-1 β), and inducible nitric oxide synthase (iNOS) were reduced after administration (Fig. 7E-H). TNF-α, IL-6 and iNOS are markers of the inflammatory state of M1 polarization, while interleukin-10 (IL-10), CD206, and Arginase-1 (Arg-1) represent the anti-inflammatory state of M2 polarization [38–40]. Collectively, M2 polarization is critical for ALI-lung attenuation of PGE2-MSC administration.

### Therapeutic mechanisms of PGE2-MSCs in LPS-ALI mice via the SMAD3, α-SMA and MMP2 pathways

The accumulation of immune cells and immune mediators such as cytokines increases the fibrotic risk of lung tissue. Therefore, we investigated the expression of SMAD3, α-smooth muscle actin (α-SMA), and matrix metalloproteinase-2 (MMP2). Our data showed that in agreement with RNA-seq analysis, PGE2-MSC treatment with PGE2-MSCs significantly downregulated SMAD3, α-SMA and MMP2 (Fig. 7A-D, Additional file 1: Fig. S4), and cytokine profiles have been previously described (Fig. 7 E–H). Experimental evidence in specific organisms can be generalized to other organisms through genomic information. To investigate the potential mechanisms of PGE2-MSCs, we applied bioinformatics tools based on RNA sequencing analysis (Fig. 5A, B, Additional file 1: Fig. S5A-D, Fig. S6). We used the GO and KEGG pathways to detect genes related to inflammation. Therefore, several pathways have been represented as potential mechanisms, such as the MAPK-NIK/NF-kappaB-TLR/JAK-STAT pathways.

## Discussion

**Fig. 8:**
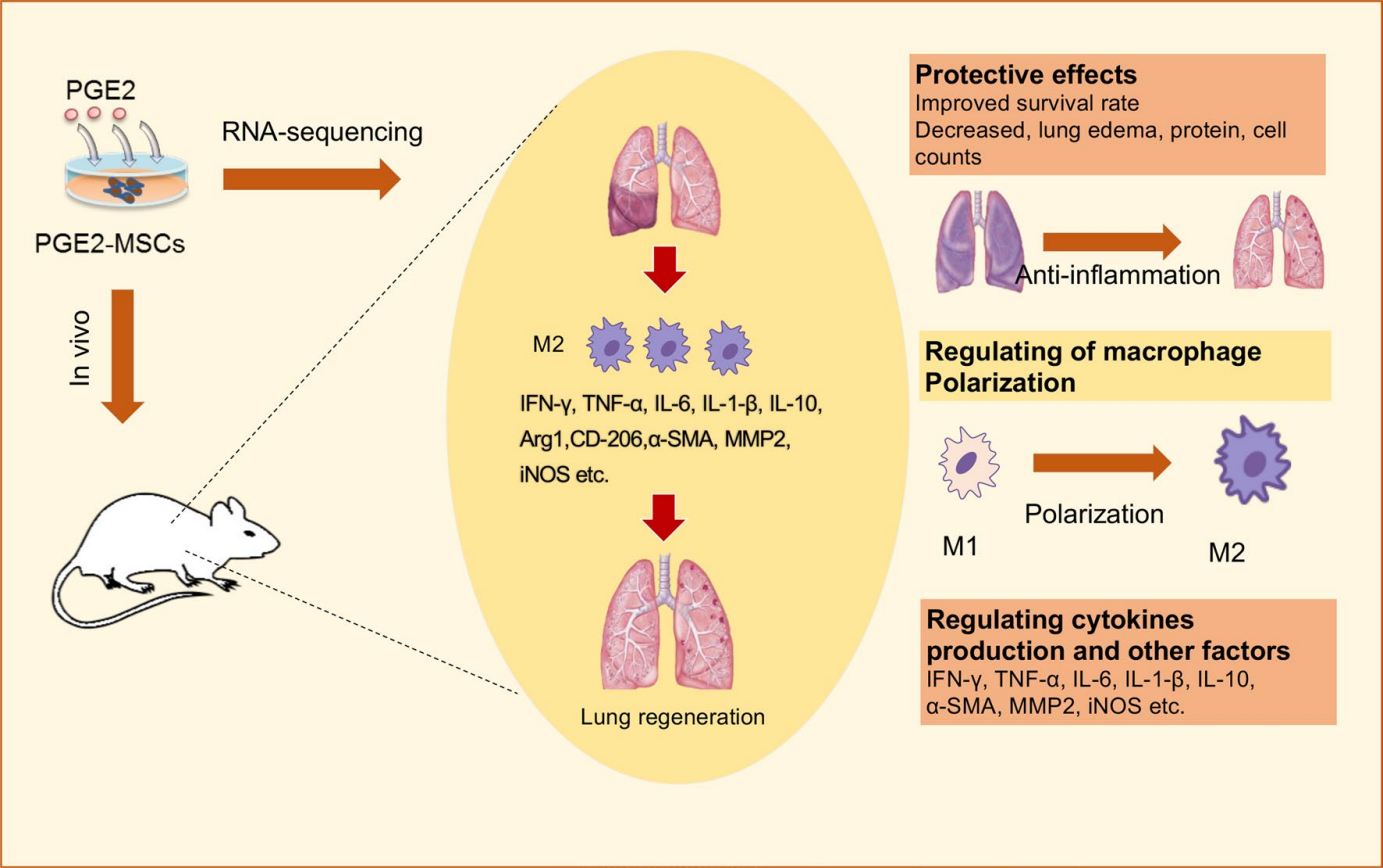
Schematic diagram of the PGE2-MSC treatment mechanisms for LPS-ALI by regulating macrophage polarization. Both RNA-seq analysis and experimental results prove that treatment with PGE2-MSCs effectively protected LPS-induced ALI induced by LPS by several mechanisms that mediate the regulation of immune cells, anti-inflammation, fluid clearance, and tissue regeneration. This scheme was created by authors

The summary of our present study is as follows: intravenous administration of PGE2-MSCs to mice successfully delivered to the lung significantly improved survival and weight ratio and obviously reduced lung inflammation, total cell number and protein permeability in LPS-induced ALI mice. The therapeutic effects of PGE2-primed MSCs were better than those of MSCs as a single treatment. Histopathological changes and immune cell findings of PGE2-MSCs support our hypothesis of the therapeutic potential of PGE2-priming MSCs, which could improve lung regeneration and mediate the balance of immune response cells in LPS-induced ALI models. The comprehensive genomic analysis provides other evidence of the therapeutic potential and molecular mechanisms of PGE2 priming MSCs for LPS-induced ALI models, and macrophage polarization plays an essential role in anti-inflammation and regeneration of the injured alveolus with clear antifibrotic efficacy in ALI mice (Fig. 8).

Macrophage polarization is thought to be a dynamic, developing, and heterogeneous phenomenon. The microenvironment affects macrophage phenotypes and functions. Regulating the production of cytokines and transcription factors may control cellular function during polarization [41, 42]. Macrophages play a critical role in the inflammatory response following ALI by releasing inflammatory mediators. Macrophage-polarized M1 macrophages are proinflammatory macrophages that can be found in the early stage of tissue injury, while M2 macrophages contribute significantly to tissue regeneration [43, 44]. In a pathological state, the expression level of proinflammatory cytokines such as IFN-γ, TNF-α IL-6 and IL-1-β increases with a reduction in macrophage-associated mediators such as IL-10 [30, 45]. Here, we found that the administration of PGE2-MSCs induced macrophages to shift toward M2 macrophages, suggesting that the critical regulator of PGE2-MSCs in tissue regeneration could be controlled by mediating macrophage polarization.

Both primary and preclinical applications of MSC-based therapy have powerfully attracted attention for the development of ARDS treatments and other lung disorders [11, 46]. By transferring several bioactive molecules, MSCs play a crucial role in physiological and pathological conditions [32, 47]. The interaction between MSCs and target cells is critical for their MSC functions [38, 48, 49]. Increasing evidence shows that the therapeutic efficacy and paracrine factors of MSCs could be influenced by biological, biochemical, and/or biophysical factors. In fact, priming of MSCs is required to improve their roles in the microenvironment of injured tissues [27, 50–52].

PGE2, a major prostaglandin generated by COX-1 and COX-2 enzymes, mediates several physiological and pathological roles [19, 53]. Several cell types can produce PGE2, such as fibroblasts and inflammatory cells, and the distinct secretion of PGE2 occurs during the immune response [54, 55]. It has been reported that PGE2 may play proinflammatory and anti-inflammatory roles by binding to EP1-EP4 receptors [35, 56, 57]. Via PGE2 secretion, MSCs mediate the activation of macrophage (M2) polarization and inhibit the proliferation of activated T, NK and NKT cells [49, 58]. Recent work using the bioactive molecule PGE2 showed promise for angiogenesis functions and regenerative therapy [35].

Different strategies have been introduced as suggestions for approaches to enhance the therapeutic potential of stem cells. Priming MSCs with biofactors and chemical factors improved the therapeutic efficacy of MSCs by regulating their secretion [27, 28]. Previous works have extensively studied the potential roles of MSC priming by a wide range of biofactors, such as IFN-γ [59–61], TNF-α [62, 63], IL-1α-β [64], FGF-2 [65], LPS [66], IL-17A [67], TLR3 [68] and IGF-1 [69, 70], which represent promising findings in improving MSC treatment profiles for different diseases. This work is the first study to use PGE2 to prepare MSCs with therapeutic potential for LPS-induced ALI models. Our previous work reported the therapeutic potential of MSC-EVs in radiation-induced lung injury and their roles in endothelial cell damage, vascular permeability, inflammation, and fibrosis [37]. At present, we reveal the protective effects of soothing and regenerating. Related work reported the therapeutic efficacy of MSCs and their extracellular vesicles for lung inflammation, including COVID-19, by secreting a wide range of paracrine factors and balancing immune response processes [71–74].

In addition to our preliminary investigation, several studies reported histopathological changes in ALI models with increasing adaptive immune cell infiltration (macrophages and neutrophils) and capillary permeability [31, 75]. Our findings showed that PGE2-primed MSCs markedly reduced the numbers of macrophages and neutrophils in BALF. As in previous reports, LPS-induced ALI leads to edema with increasing protein concentration and total cell numbers in BALF [76, 77], and our treatment with PGE2 priming MSCs evidently reduced both in BALF. Inflammatory regulators, especially macrophage-associated cytokines, upregulate inflammatory reactions and are known as the etiology of the cytokine storm, which is a considerable obstacle for lung treatments. They also represent the hallmark of acute lung injury [31, 78, 79]. Using genomic, proteomic and experimental analyses, we observed that PGE2-primed MSCs show a marked ability to balance proinflammatory and anti-inflammatory cytokines.

Our strategy has some limitations. First, this work achieved acute lung injury, but ALI can progress to ARDS in many cases and cause substantial respiratory system failure. Delaying the transition from ALI to ARDS should therefore be considered as well. In addition, we employed a single dose of MSCs and PGE2-MSCs that was set arbitrarily and with the help of prior experience. Dose‒response studies, which compare the effects of varying doses of MSCs and PGE2-MSCs, should be a standard part of future research to find optimal and minimally effective doses. Finally, we described the genomic and molecular mechanisms contributing to the protective effects of MSCs and PGE2-MSCs. Nevertheless, it would be clinically and pharmacologically useful to investigate which small molecules and RNAs are the functional components mediating the protective effects of PGE2-MSCs.

## Conclusion

In summary, this work was designed to use PGE2 priming to enhance the therapeutic potential of MSCs for ALI models. Our findings revealed that PGE2-primed MSCs conclusively protected and regenerated lung injury after LPS challenge. PGE2 priming of MSCs elevates the polarization of macrophages from the proinflammatory subset (M2) to the anti-inflammatory subset (M2) by regulating cytokine production and blocking polymorphonuclear neutrophil influx into the injured tissue and preventing further damage. Therefore, M2 macrophage polarization is a critical mediator of PGE2 priming of MSCs in ALI models. This study provides a candidate for ALI treatment and deserves attention in future clinical settings.

## Supplementary Information

**Additional file 1. Table S1**. Complete details of antibodies. **Table S2**. Primers Used for Real-Time PCR. **Fig. S1**. Dose and time point effects of LPS-induced ALI in mouse models. **Fig. S2**. RNA-Seq analysis to assess the expression patterns of differentially expressed genes under different experimental conditions. **Fig. S3**. PGE2-MSCs regulate macrophage polarization and cytokine production. **Fig. S4**. Images of the uncropped immunoblots are shown in Fig. 7A.

## Data Availability

The transcriptome sequence data have been deposited in the NCBI Sequence Read Archive under the primary accession code PRJNA938188 (https://www.ncbi.nlm.nih.gov/bioproject/PRJNA938188). All other data are included in the article and its Supplementary Information files or available from the corresponding authors upon reasonable request.

## Abbreviations

ALI: Acute lung injury
ARDS: Acute respiratory distress syndrome
BLI: Bioluminescence imaging
GFP: Green fluorescent protein
eGFP: Enhanced green fluorescence protein
Fluc: Firefly luciferase
hP-MSCs: Human placental Mesenchymal stem cell
MSCs: Mesenchymal stem cells
BCA: Bicinchoninic acid
COVID-19: Coronavirus disease 2019
FBS: Fetal bovine serum
H&E: Hematoxylin and eosin
PBS: Phosphate-buffered saline
PCR: Polymerase chain reaction
WB: Western blot
PVDF: Polyvinylidene difluoride
