# Supplementary material for: Superior protective effects of PGE2 priming mesenchymal stem cells against LPS-induced acute lung injury (ALI) through macrophage immunomodulation
Source: Stem Cell Res Ther. 2023 Mar 22;14:48. doi: 10.1186/s13287-023-03277-9 (PMC10032272; doi:10.1186/s13287-023-03277-9)
Supplement: Supplementary file 1 — Additional file 1. Table S1. Complete details of antibodies. Table S2. Primers Used for Real-Time PCR. Fig. S1. Dose and time point effects of LPS-induced ALI in mouse models. Fig. S2. RNA-Seq analysis to assess the expression patterns of differentially expressed genes under different experimental conditions. Fig. S3. PGE2-MSCs regulate macrophage polarization and cytokine production. Fig. S4. Images of the uncropped immunoblots are shown in Fig. 7A. [file 13287_2023_3277_MOESM1_ESM.docx]

**Supplementary data**

**Supplementary Table S1.** **Complete details of antibodies**

| Antibodies | Application | Source | Identifier | Dilution |
| --- | --- | --- | --- | --- |
| Anti-F4/80 | IF | Proteintech | 28463-1-AP | 1:200 |
| Anti-CD206 | IF | Proteintech | 18704-1-AP | 1:200 |
| Anti-MMP2 | WB | Kleanab | P106566 | 1:2000 |
| Anti-smad3 | WB | Proteintech | 66516-1-Ig | 1:2500 |
| Anti-a-SMA | WB | Boster | BM3902 | 1:2000 |
| Anti-GAPDH | WB | Proteintech | 60004-1-Ig | 1:20000 |

**Supplementary Table S2. Primers Used for Real-Time PCR**

| NAME | Primers |
| --- | --- |
| TNF-α | Forward: ACGGCATGGATCTCAAAGAC  Reverse: AGATAGCAAATCGGCTGACG |
| IL-1Β | Forward: GCAACTGTTCCTGAACTCAACT  Reverse: ATCTTT GGGGT CCGTCAACT |
| IL-6 | Forward: TAGTCCT TCC TACCCCAATTTCO  Reverse: TTGGTCCT TTAGCCACTCCT TTC |
| IL-10 | Forward: GCTCTTACTTGACTGGCATGAG  Reverse: CGCAGCTCTAGGAGCATGTG |
| CD206 | Forward: CTCTGTTCAGCTATTGGACGC  Reverse: CGGAATTTTCTGGGATTCAGCTTC |
| Arg-1 | Forward:CTCCAAGCCAAAGTCCTTTAGAG  Reverse: AGGAGCTGTCATTAGGGACATC |
| iNOS | Forward:GTTCTCAGCCCAACAATACAAGA  Reverse: GTGGACGGGTCGA TGTCAC |
| GADPH | Forward: AGGTCGGTGTGAACGGATTTG  Reverse:TGTAGACCATGTAGTTGAGGTCA |

**
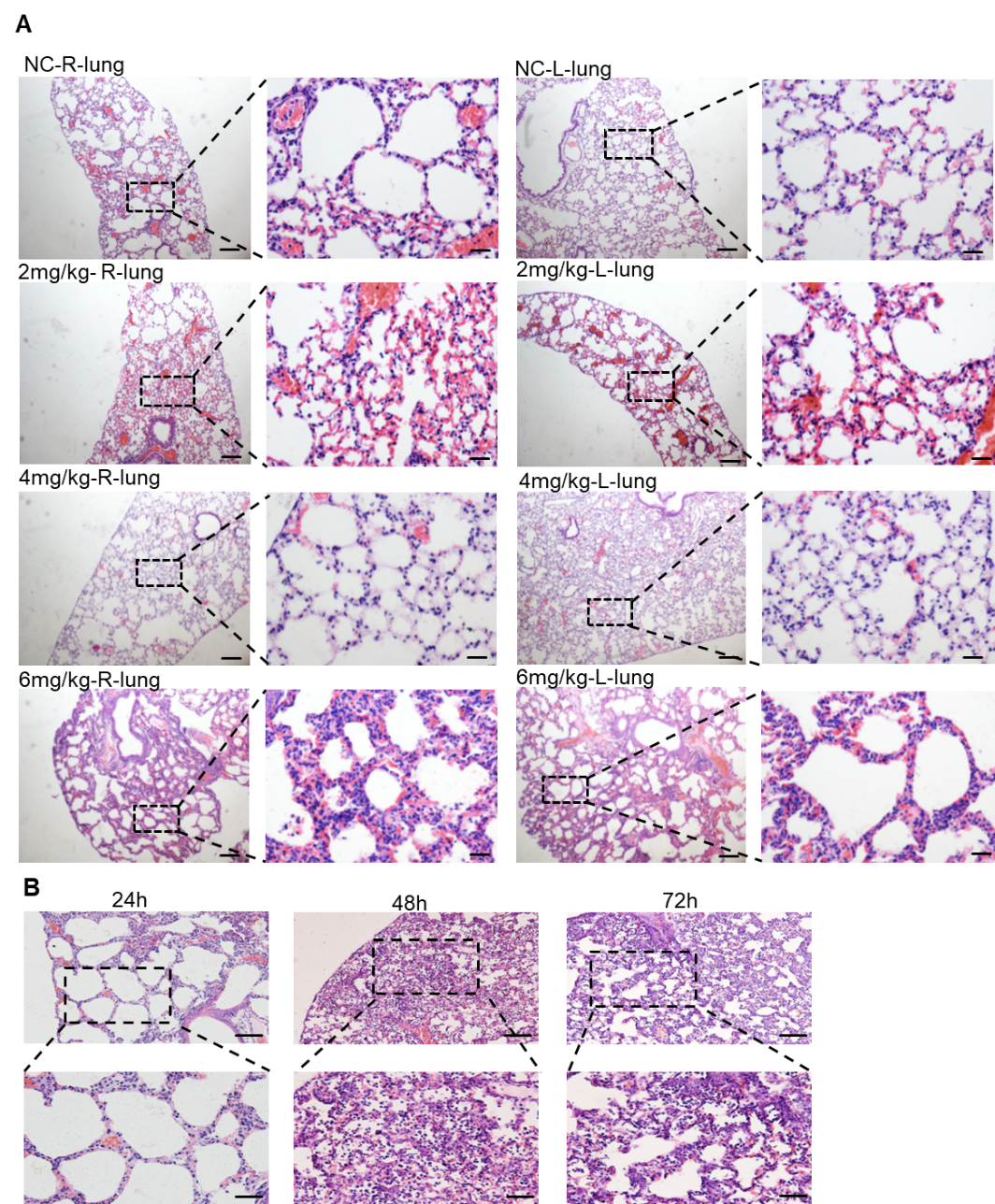
SUPPLEMENTAL FIGURES & LEGENDS**

**Supplementary Fig. 1. Dose and time point effects of LPS-induced ALI in mouse models.**  (**A**) Represent the different doses of LPS to establish animal model candidates for further investigations. LPS (6 mg/kg) can induce obvious lung injury, but has a high fatality rate. In present study, LPS (5 mg/kg) was applied. (**B**) Represent the LPS effects in ALI models during different time points and results revealed the severity of inflammation at 48h. NC, normal control; R, right; L, left.


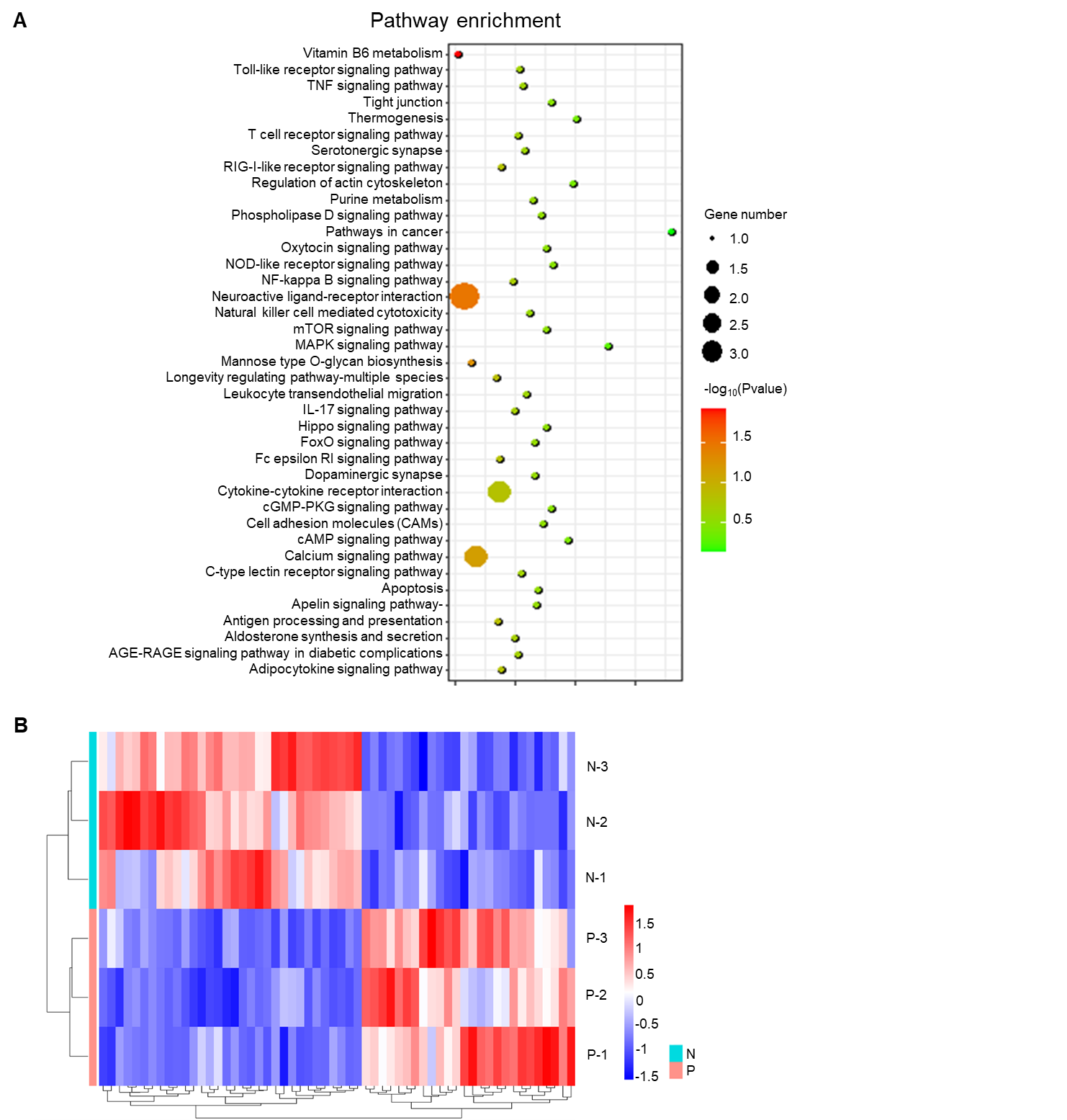


**Supplementary Fig. 2. RNA-Seq analysis to assess the expression patterns of differentially expressed genes under different experimental conditions.** (A) Kyoto Encyclopedia of Genes and Genomes (KEGG) enrichment analysis to determine which differentially expressed genes were significantly enriched compared with the whole genome background to determine the main biological functions exercised by the differentially expressed genes. (B) Heatmap to perform bidirectional clustering analysis of differential genes and samples of all comparison groups, clustering according to the expression level of the same gene in different samples and the expression patterns of different genes in the same sample, using Euclidean. The method calculates the distance, and the hierarchical clustering longest distance method (complete linkage) is used for clustering (N=MSCs, P= PGE2-MSCs).

| 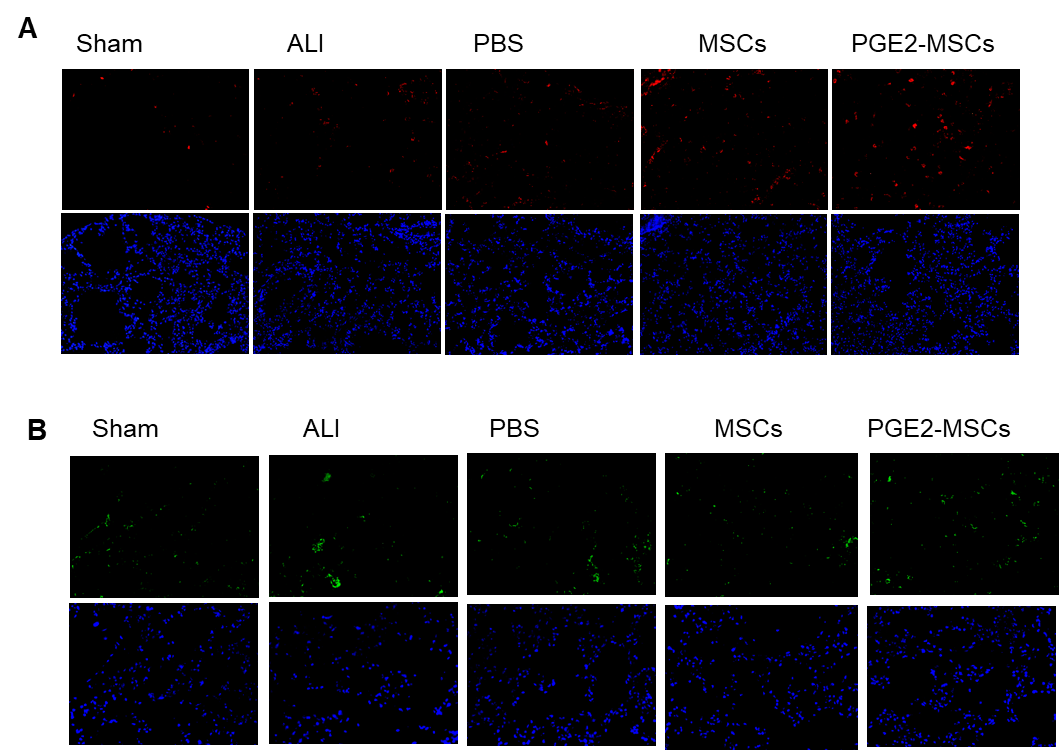 |
| --- |
| **Supplementary Fig. 3.** PGE2-MSCs regulate macrophage polarization and cytokine production. **(A)** Representative immunofluorescence images of F/4/80 expression (red) in LPS-induced ALI mice. **(B)** Immunofluorescence images of CD206 expression (green) in LPS-induced ALI mice. Merge images can be found in **Fig. 6**. |
| 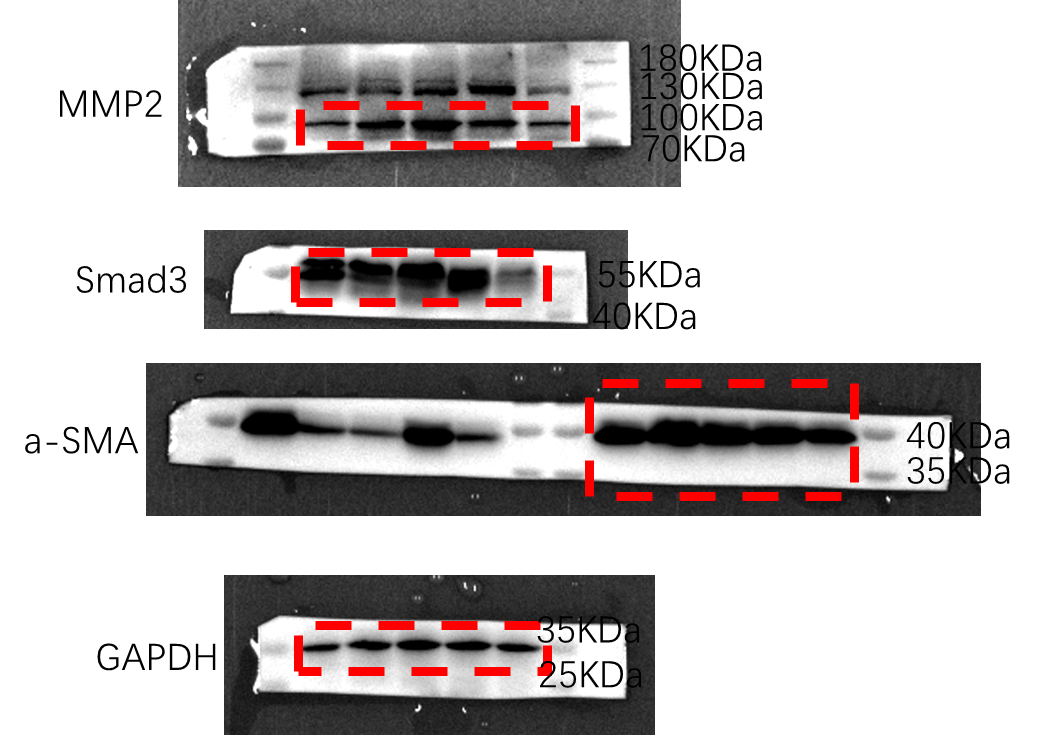 |

**Supplementary Fig. 4. Images of the uncropped immunoblots shown in Fig. 7A. Boxes indicate cropped regions.**

**
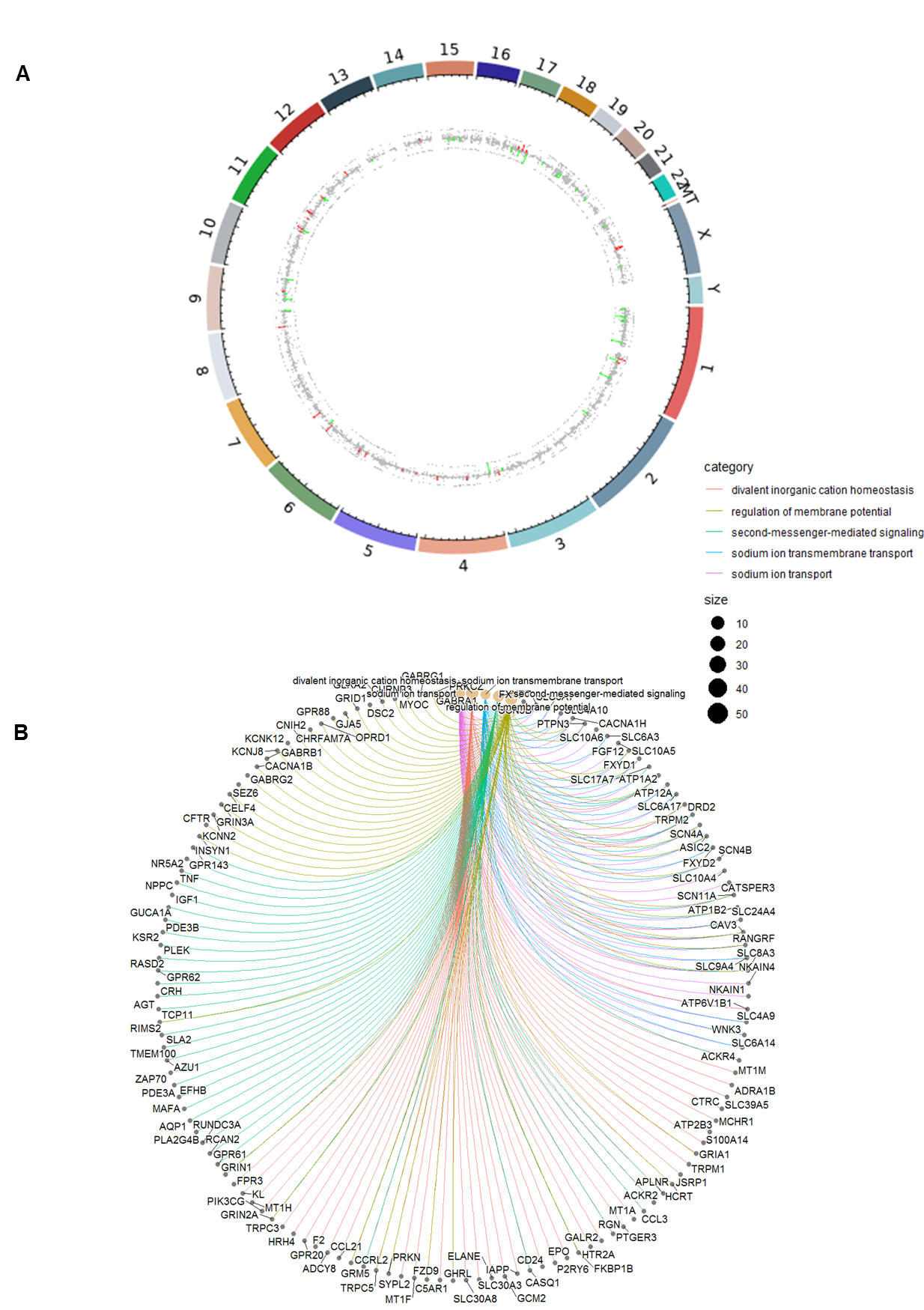
**

**
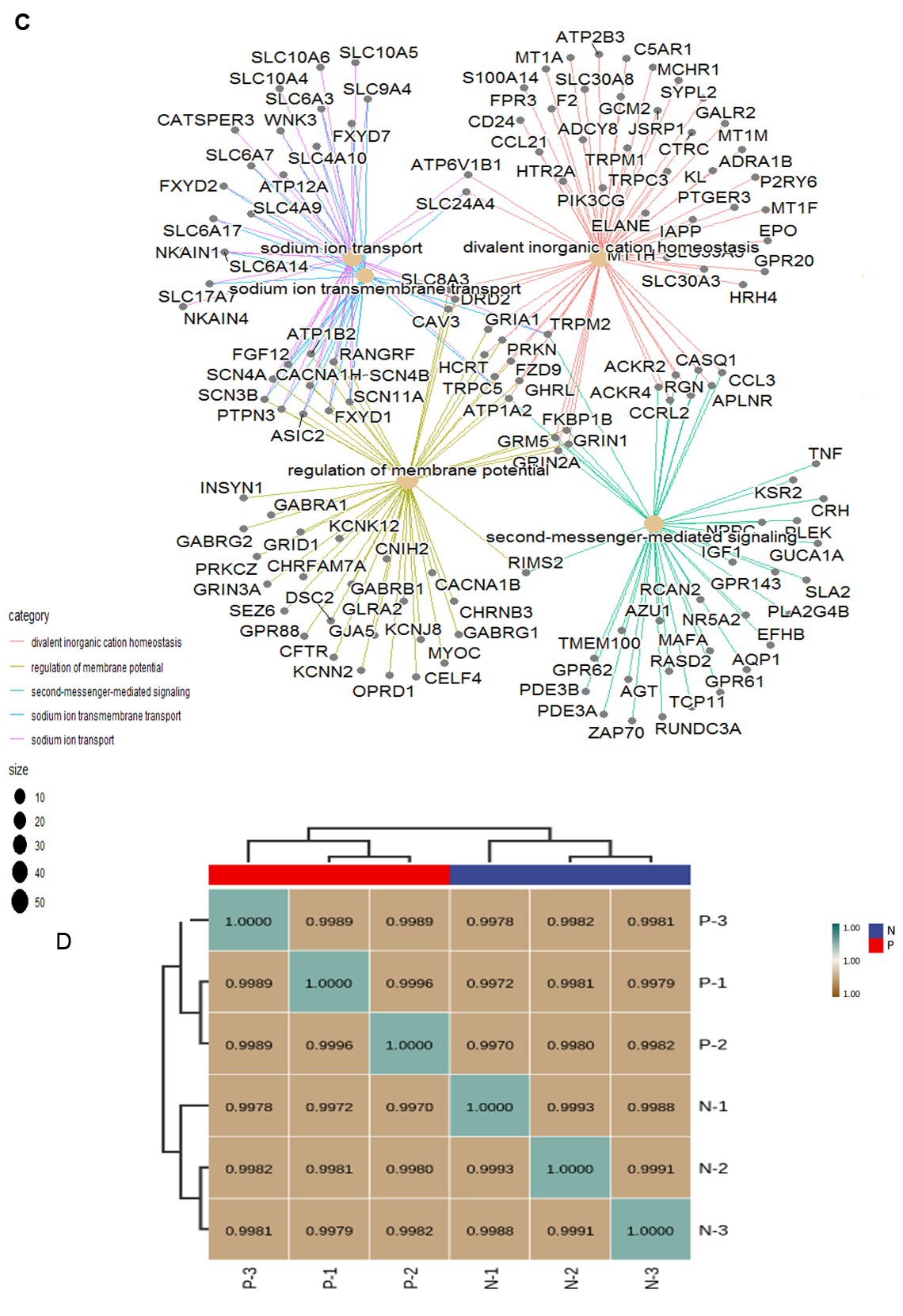
**

**Supplementary Fig. 5. RNA differential expression analysis results.** (**A**) Genome circle map. The differentially expressed RNAs are marked on the genome to draw a genome circle diagram, which can reflect the distribution of differentially expressed genes between different comparison groups on chromosomes. The outermost circle is the chromosome band, and from the outside to the inside are the differential expression analysis results of different differential analyses. Red and green are histograms of log2FoldChange values of up- and downregulated genes, respectively, and gray are scatterplots of log2FoldChange values of nondifferentially expressed genes. (**B-C**) The gene-concept network of functional GO enrichment results. The connection between a gene and a term means that the gene is involved in this GO term. (**D**) Represents the correlation of gene expression levels between samples. The closer the correlation coefficient is to 1, the higher the similarity of expression patterns between samples. The correlation between 0.8 and 1 is a very strong correlation, while less than 0.8 represents a low correlation.

**
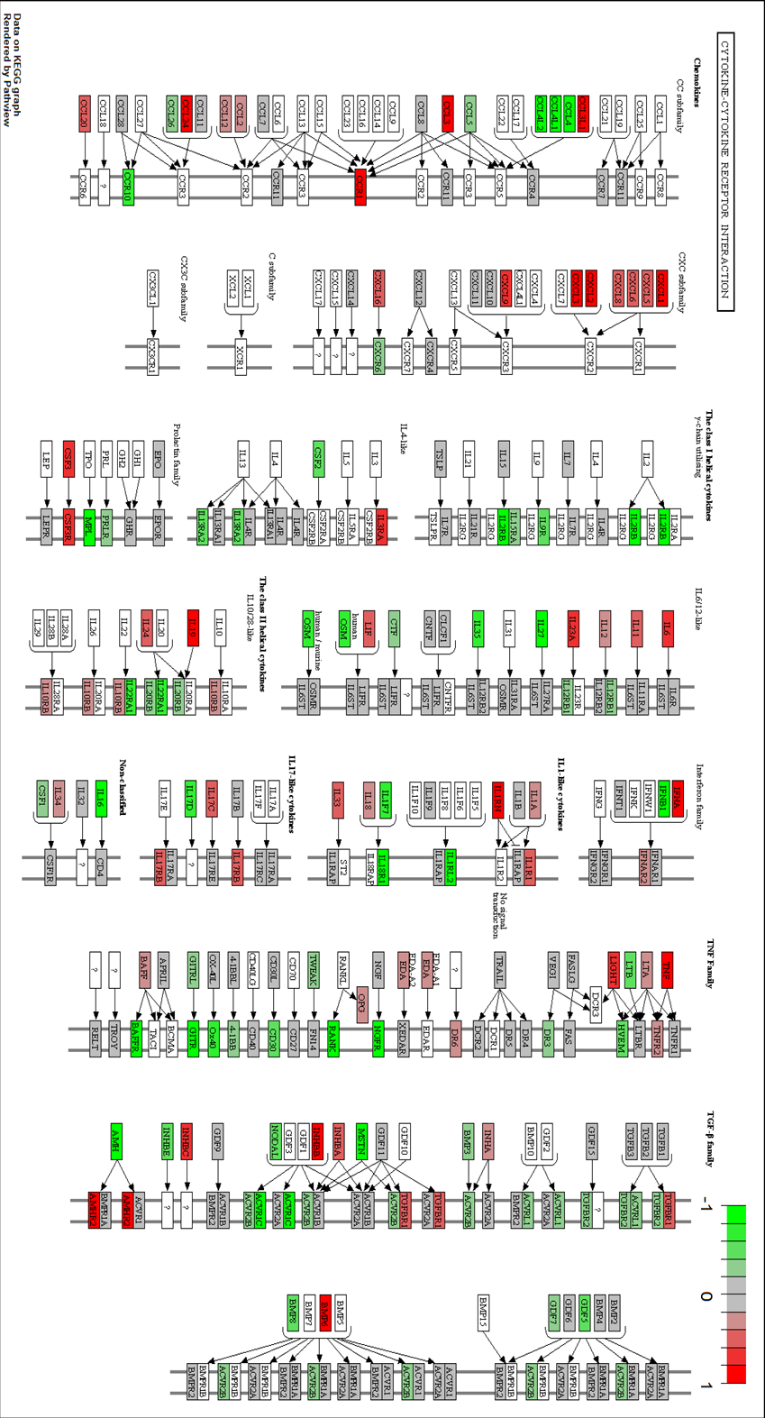
Supplementary Fig. 6.** Pathview of the cytokine‒cytokine receptor interaction pathway. Node color change reflects log fold-change (FC) range
